# Automated segmentation of cerebral deep gray matter from MRI scans: effect of field strength on sensitivity and reliability

**DOI:** 10.1186/s12883-017-0949-4

**Published:** 2017-09-05

**Authors:** Renxin Chu, Shelley Hurwitz, Shahamat Tauhid, Rohit Bakshi

**Affiliations:** 1 Laboratory for Neuroimaging Research, Brigham and Women’s Hospital, Harvard Medical School, 60 Fenwood Rd, Mailbox 9002L, Boston, MA 02115 USA; 2 Departments of Neurology, Brigham and Women’s Hospital, Harvard Medical School, Boston, MA USA; 3 Radiology, Brigham and Women’s Hospital, Harvard Medical School, Boston, MA USA; 4 Partners MS Center, Brigham and Women’s Hospital, Harvard Medical School, Boston, MA USA; 5 Department of Medicine, Brigham and Women’s Hospital, Harvard Medical School, Boston, MA USA

**Keywords:** Multiple sclerosis, Subcortical deep gray matter, Atrophy, 3T MRI, Brain segmentation

## Abstract

**Background:**

The cerebral subcortical deep gray matter nuclei (DGM) are a common, early, and clinically-relevant site of atrophy in multiple sclerosis (MS). Robust and reliable DGM segmentation could prove useful to evaluate putative neuroprotective MS therapies. The objective of the study was to compare the sensitivity and reliability of DGM volumes obtained from 1.5T vs. 3T MRI.

**Methods:**

Fourteen patients with MS [age (mean, range) 50.2 (32.0–60.8) years, disease duration 18.4 (8.2–35.5) years, Expanded Disability Status Scale score 3.1 (0–6), median 3.0] and 15 normal controls (NC) underwent brain 3D T1-weighted paired scan-rescans at 1.5T and 3T. DGM (caudate, thalamus, globus pallidus, and putamen) segmentation was obtained by the fully automated FSL-FIRST pipeline. Both raw and normalized volumes were derived.

**Results:**

DGM volumes were generally higher at 3T vs. 1.5T in both groups. For raw volumes, 3T showed slightly better sensitivity (thalamus: *p* = 0.02; caudate: *p* = 0.10; putamen: *p* = 0.02; globus pallidus: *p* = 0.0004; total DGM: *p* = 0.01) than 1.5T (thalamus: *p* = 0.05; caudate: *p* = 0.09; putamen: *p* = 0.03; globus pallidus: *p* = 0.0006; total DGM: *p* = 0.02) for detecting DGM atrophy in MS vs. NC. For normalized volumes, 3T but not 1.5T detected atrophy in the globus pallidus in the MS group. Across all subjects, scan-rescan reliability was generally very high for both platforms, showing slightly higher reliability for some DGM volumes at 3T. Raw volumes showed higher reliability than normalized volumes. Raw DGM volume showed higher reliability than the individual structures.

**Conclusions:**

These results suggest somewhat higher sensitivity and reliability of DGM volumes obtained from 3T vs. 1.5T MRI. Further studies should assess the role of this 3T pipeline in tracking potential MS neurotherapeutic effects.

## Background

The cerebral subcortical deep gray matter nuclei (DGM) are a common and clinically-relevant site of atrophy, beginning in the early stages of multiple sclerosis (MS) [1–4]. In addition, DGM atrophy is a feature of progressive forms of the disease and can be shown to progress in as little as 1 year [5, 6]. Given that few treatments are available for patients with progressive forms of MS [7], this represents a major unmet need, calling for the availability of new outcome measures to screen putative therapies. MRI-defined cerebral lesion activity and total burden, traditionally used in trials of patients with relapsing forms of MS [8–10], are less sensitive to change in patients with advanced disability and progressive forms of the disease [11, 12]. In one study, DGM atrophy assessment over 1 year was successful in demonstrating a treatment effect in MS [5]. Therefore, robust, sensitive, and reliable segmentation of DGM structures could prove useful in the evaluation of new MS therapies in all stages of the disease [6].

Field strength is known to bias the sensitivity and detectability of global cerebral MRI-based assessments of lesions and atrophy in MS [13–15]. The most commonly available MRI platforms employed for routine clinical care and research investigations are 1.5T and, less commonly, 3T. To date, it has not been clear whether longitudinal cerebral atrophy determinations would benefit from higher field strength acquisitions. The purpose of this study was to employ a fully automated freely available segmentation pipeline to compare the sensitivity and reliability of DGM volumetrics obtained from 1.5T vs. 3T MRI scans in normal controls (NCs) and patients with MS.

## Methods

### Subjects and neurologic examination

Fourteen patients with MS and 15 normal controls (NCs) were recruited to undergo brain MRI at both 1.5T and 3T. Table 1 shows the demographic and clinical characteristics of the subjects. The two groups differed on age (*p* = 0.0009) but not sex (*p* = 0.43). Patients met the International Panel criteria for either MS or a clinically isolated demyelinating syndrome (CIS) [16]. All patients underwent an examination by an MS specialist neurologist including evaluation of the Expanded Disability Status Scale (EDSS) [17] score and timed 25-ft walk (T25FW) [18]. At the time of MRI, 10 patients were on disease modifying therapy (DMT), while 4 patients were not. Among the DMTs used, four subjects were on dimethyl fumarate, three were on natalizumab, and one each were on fingolimod, glatiramer acetate, or cyclophosphamide. None of the DMTs were started in the 3 months before MRI. Our hospital’s human research ethics board (The Partners Human Research Committee) approved this study and written informed consent was obtained on all subjects. This work was presented in preliminary form at the 2015 annual meeting of the European Committee on Treatment and Research in Multiple Sclerosis (ECTRIMS), Barcelona, Spain; and at the 2016 annual meeting of the American Academy of Neurology, Vancouver, Canada.

**Table 1:** Demographic and clinical characteristics

	MS (*n* = 14)	NC (*n* = 15)
Sex ratio (women/men)^a^	0.79 (11/3)	0.60 (9/6)
Age, years^a^	50.2 ± 8.2 (32–60)	37.7 ± 9.6 (25–52)
MS disease category, n (%)		
-Progressive relapsing MS	1 (7%)	–
-Secondary progressive MS	3 (21%)	–
-CIS or relapsing-remitting MS	10 (71%)	–
Disease duration, years^b^	18.4 ± 10.7 (8.2–35.5)	–
EDSS score	3.1 ± 2.1 (0–6) (median 3.0)	–
Timed 25-ft walk, seconds	6.2 ± 2.7 (3.5–13.0)	–

Key: Data are mean ± standard deviation (range) unless otherwise indicated; *MS* multiple sclerosis, *NC* normal controls, *CIS* clinically isolated demyelinating syndrome, *EDSS* Expanded Disability Status Scale; ^a^MS vs. NC were different on age (*p* = 0.0009) but not on sex (*p* = 0.43); ^b^Time from first symptoms; n = number of subjects

### MRI acquisition

All subjects underwent brain MRI at 1.5T (Signa; General Electric, Milwaukee, WI) and 3T (Skyra; Siemens, Erlangen, Germany). Scanner and acquisition details are show in Table 2. On both platforms, we obtained high-resolution 3D T1-weighted sequences covering the whole head. These were matched as closely as possible on voxel size and acquisition time, considering practical scan time limits for patient tolerability. Each sequence was optimized for signal-to-noise based on previous clinically-routine development. Each subject had a scan followed by a re-scan on the same day on each platform. Thus, at each field strength, two scans were acquired from each subject, where the subject was removed from the scanner between scans for a few minutes, and was repositioned and rescanned by the MRI technologist. For all subjects, except three, the 1.5T and 3T imaging was performed on the same day for a given subject. For the remaining three subjects, the interval between 1.5T and 3T acquistion was 6, 16, or 47 days. During the study period, there were no intervening scanner upgrades.

**Table 2:** 1.5T and 3T brain MRI acquisition protocols

	1.5T	3T
Scanner manufacturer	GE Signa LX	Siemens Skyra
Operation system version	11×	D13
Coil	Quadrature head coil	20-channel head and neck coil
Type of sequence	3D SPGR	3D MPRAGE
Acceleration factor for parallel imaging	N/A	2
Orientation	Sagittal	Sagittal
Field of view (cm)	24 × 24	24 × 25.6
Matrix size	256 × 256	240 × 256
Number of slices	166	176
Repetition time (msec)	8.176	2300
Echo time (msec)	3.856	2.96
Flip angle (degrees)	20	9
Voxel size (mm)	0.938 × 0.938 × 1.2	1.0 × 1.0 × 1.0
Scan time (minutes)	6:24	5:12
Number of signal averages	1	1

Key: *SPGR* spoiled gradient recalled echo, *MPRAGE* magnetization-prepared rapid acquisition gradient echo

### MRI analysis

**Fig. 1:**
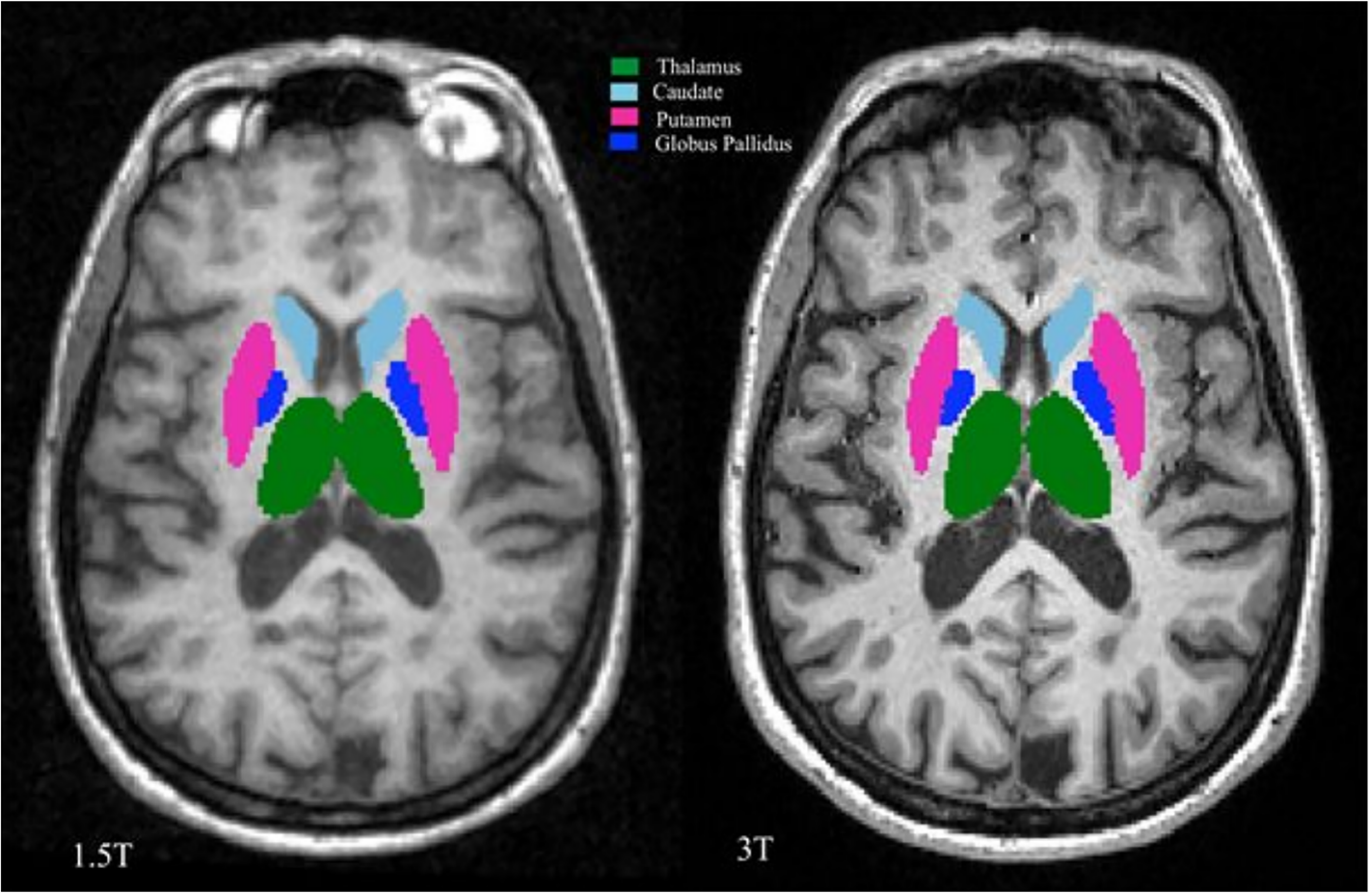
Representative anatomic slice showing segmentation of the cerebral subcortical deep gray matter (DGM) in one patient from 1.5T (left) and 3T (right) MRI scans. This is from a 51 year-old woman with multiple sclerosis and moderate physical disability. The total DGM volume was 28.4 ml at 1.5T and 29.3 ml at 3T. Component DGM structures are shown in different colors. The segmentation maps are overlaid to the original raw 3D T1-weighted images after re-sampling to the axial plane. Segmentation was performed by the fully automated FSL-FIRST pipeline. In the present study, we utilized the FSL-FIRST outputs to assess the volume of the thalamus, caudate, putamen, and globus pallidus (and their sum = total DGM)

All image pre-processing was performed using Jim software (v.7.0, Xinapse Systems Ltd., Northants, UK, http://www.xinapse.com/). For both the 1.5T and 3T images, the raw sagittal images did not yield adequate segmentation, particularly of the intracranial volume (ICV) cavity (“skull stripping”; data not shown). With optimization work, we determined necessary pre-processing steps, which were the same for the 1.5T and 3T images. First, all original DICOM images were converted to a Neuroimaging Informatics Technology Initiative (NIfTI) format, and their raw sagittal orientation was converted to axial. Then, 170 axial slices were extracted from each scan starting at the first slice showing the top of the head. This provided whole brain coverage in all patients extending to the foramen magnum. DGM (caudate, thalamus, globus pallidus, and putamen) volumes were obtained by a fully automated segmentation pipeline (FSL-FIRST, v. 5.0, The Analysis Group, Oxford, UK, http://fsl.fmrib.ox.ac.uk/fsl/fslwiki/FIRST) (Fig. 1). This pipeline was chosen for its free availability, full automation, and utility shown in detecting short term DGM atrophy and treatment effects in patients with MS [5, 6]. The ICV was the sum of gray matter, white matter and cerebrospinal fluid (CSF), which was obtained by applying these images to a fully automated algorithm (SIENAX, v. 5.0, The Analysis Group, Oxford, UK, http://fsl.fmrib.ox.ac.uk/fsl/fslwiki/SIENA) [15, 19]. We assessed three volumetric measures of the DGM structures: 1) raw volumes, 2) those that were normalized by dividing by the subject’s ICV (“fractions” [1]); 3) those that were normalized by multiplying the raw volume by the whole brain SIENAX normalization factor (“normalized”).

### Statistical Analysis

Statistical analyses included unpaired and paired t-tests, Fisher’s exact tests, and analysis of covariance with age as a covariate. Correlations were reported for MRI associations with age, EDSS score, and T25FW by Spearman coefficients. Sensitivity (differentiation between groups) was reported using mixed model analysis of covariance with age as covariate, comparing the methods for their ability to differentiate MS from NC by the interaction between method and group. Within-subject correlations were compared using the method of Meng et al. (1992) [20]. Reliability was reported using intraclass correlation coefficients (ICCs) [21] with 95% confidence intervals (CIs). The analysis was generated using SAS (v. 9.4, SAS Institute Inc., Cary, NC, http://www.sas.com/).

## Results

### Scan-rescan reliability: 1.5T vs. 3T

**Fig. 2:**
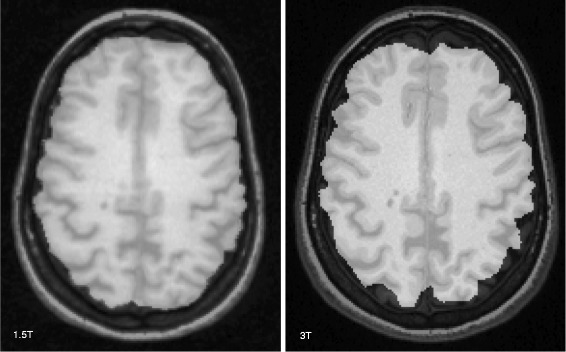
Examples of brain extraction masks obtained in the fully automated SIENAX pipeline, part of the process to determine intracranial volume. Both images are from a 51 year-old woman with multiple sclerosis and moderate physical disability. The brain parenchymal mask was more accurately obtained at 1.5T, whereas it is underestimated at 3T

DGM scan-rescan reliability within groups, comparing field strengths, is shown in Table 3 for all three methods. Reliability was generally very high for both the 1.5T and 3T measurements. Regarding raw volumes, there was perhaps somewhat higher reliability at 3T and, across all comparisons, higher reliability for measuring total DGM than the individual structures. Regarding fractions and normalized volumes, there was a suggestion of higher reliability at 1.5T for the caudate. Comparing the three methods, there was slightly higher reliability for measuring raw volumes than fractions or normalized volumes. We determined that the latter were more reliable at 1.5T most likely due to increased accuracy vs. 3T in whole brain extraction, necessary for determination of the ICV and brain size (Fig. 2).

**Table 3:** Deep gray matter data: scan-rescan reliability (within group and field strengths)

	MS (*n* = 14)		NC (*n* = 15)	
	1.5T ICC (95% CI)	3T ICC (95% CI)	1.5T ICC (95% CI)	3T ICC (95% CI)
Volumes				
-Thalamus	0.99 (0.96, 1.00)	0.99 (0.98, 1.00)	0.95 (0.87, 0.98)	0.99 (0.97, 1.00)
-Caudate	0.99 (0.98, 1.00)	0.99 (0.98, 1.00)	0.97 (0.93, 0.99)	0.96 (0.88, 0.98)
-Putamen	0.94 (0.85, 0.98)	0.97 (0.90, 0.99)	0.95 (0.87, 0.98)	0.97 (0.93, 0.99)
-Globus pallidus	0.94 (0.83, 0.98)	0.98 (0.95, 0.99)	0.95 (0.87, 0.98)	0.98 (0.93, 0.99)
-Total DGM	0.99 (0.97, 1.00)	1.00 (0.99, 1.00)	0.98 (0.94, 0.99)	0.99 (0.98, 1.00)
Fractions				
-Thalamus	0.98 (0.95, 0.99)	0.95 (0.87, 0.98)	0.88 (0.71, 0.96)	0.93 (0.81, 0.97)
-Caudate	0.98 (0.94, 0.99)	0.94 (0.84, 0.98)	0.96 (0.88, 0.98)	0.88 (0.69, 0.95)
-Putamen	0.92 (0.78, 0.97)	0.92 (0.78, 0.97)	0.93 (0.81, 0.97)	0.95 (0.86, 0.98)
-Globus pallidus	0.93 (0.80, 0.97)	0.95 (0.85, 0.98)	0.93 (0.81, 0.97)	0.85 (0.63, 0.94)
-Total DGM	0.98 (0.93, 0.99)	0.93 (0.80, 0.97)	0.94 (0.84, 0.98)	0.89 (0.72, 0.96)
Normalized				
-Thalamus	0.95 (0.87, 0.98)	0.99 (0.96, 0.99)	0.96 (0.89, 0.99)	0.96 (0.88, 0.98)
-Caudate	0.97 (0.90, 0.99)	0.98 (0.95, 0.99)	0.97 (0.93, 0.99)	0.92 (0.78, 0.97)
-Putamen	0.94 (0.83, 0.98)	0.97 (0.92, 0.99)	0.93 (0.81, 0.97)	0.95 (0.86, 0.98)
-Globus pallidus	0.92 (0.77, 0.97)	0.97 (0.92, 0.99)	0.97 (0.92, 0.99)	0.93 (0.82, 0.98)
-Total DGM	0.95 (0.87, 0.98)	0.98 (0.95, 0.99)	0.97 (0.93, 0.99)	0.95 (0.85, 0.98)

Key: *MS* multiple sclerosis, *NC* normal controls, *DGM* cerebral subcortical deep gray matter, *CI* confidence interval, *ICC* intraclass correlation coefficient; normalized = raw volume multiplied by SIENAX normalization factor

### DGM results: 1.5T vs. 3T

**Fig. 3:**
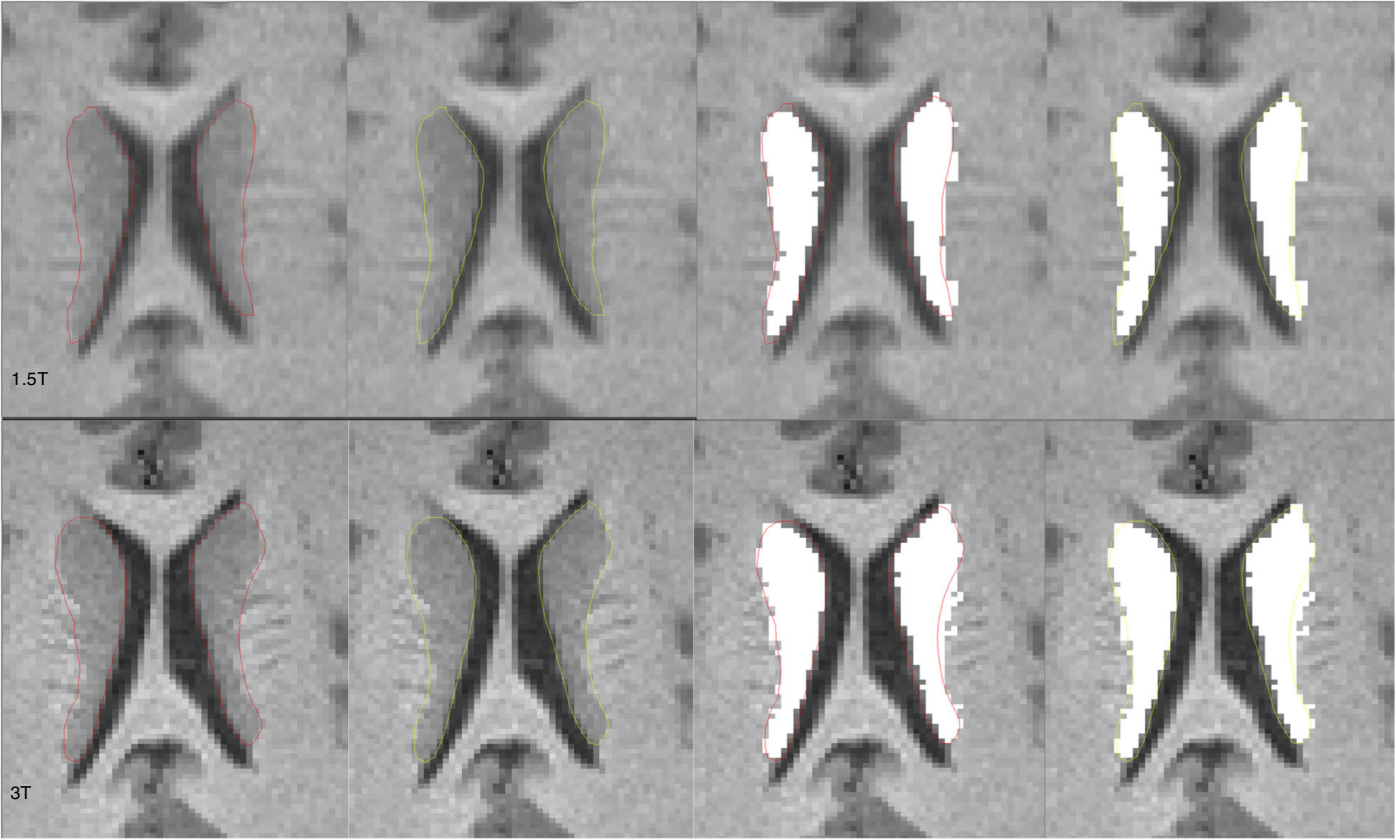
Manual (expert) segmentation overlaid with automated segmentation output (white areas) of the caudate nucleus in a healthy 33 year-old control subject from 1.5T (upper row) and 3T (lower row) 3D T1-weighted MRI scans. The total DGM volume was 36.6 ml at 1.5T and 38.9 ml at 3T. For illustrative purposes, the ground truth contours are shown in both red and yellow colors. Automated segmentation was performed by the fully automated FSL-FIRST pipeline. The 3T automated output typically provided a larger and more accurate contour than 1.5T. At 1.5T, the interface between the caudate and the medial aspects of the ventricular CSF is underestimated vs. 3T. The interface between the caudate and the adjacent (lateral) white matter appears to be captured similarly at both field strengths

**Fig. 4:**
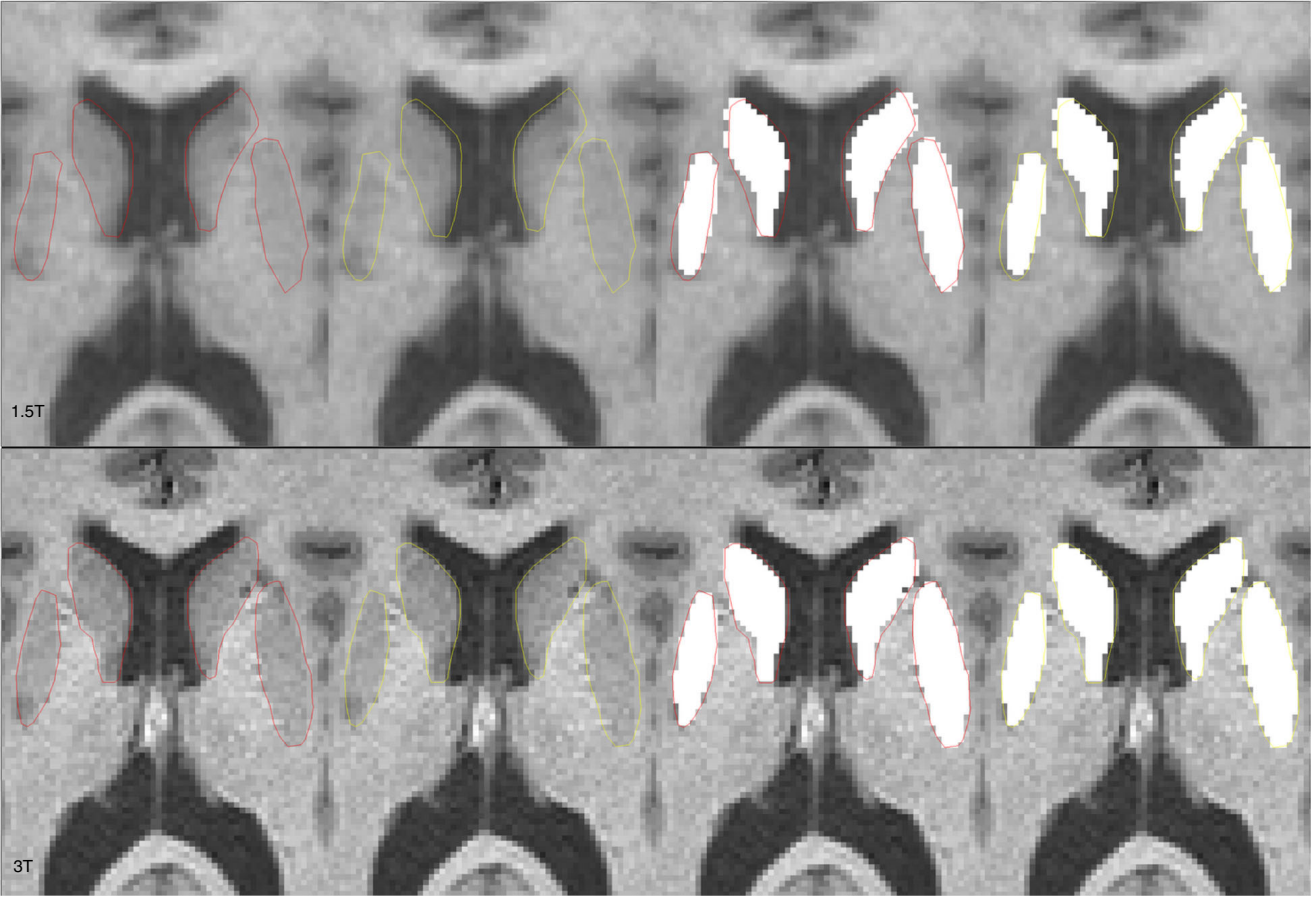
Manual (expert) segmentation overlaid with automated segmentation output (white areas) of the caudate nucleus and putamen from 1.5T (upper row) and 3T (lower row) 3D T1-weighted MRI scans. Images are from a 51 year-old woman with multiple sclerosis and moderate physical disability. The total DGM volume was 28.4 ml at1.5T and 29.3 ml at 3T. For illustrative purposes, the ground truth contours are shown in both red and yellow colors. Automated segmentation was performed by the fully automated FSL-FIRST pipeline. The 3T automated output typically provided a larger and more accurate contour than 1.5T. At 1.5T, the interface between the caudate and the anterior and medial aspects of the ventricular CSF is underestimated vs. 3T. The interface between the putamen and the adjacent (lateral) white matter appears to be larger and more accurate at 3T

Using all three methods, DGM volumes were higher at 3T than 1.5T in both patients and controls (Tables 4 and 5). This was seen across all DGM structures examined, and significant for all comparisons (except thalamic fractions in the MS group). The effect sizes were generally larger for this field strength difference for raw volumes than fractions (Tables 4 and 5) or normalized volumes (Table 5). This effect was also reflected in total DGM data. For example, the total raw DGM volume was, on average, 3.9% higher in patients and 5.9% higher in controls (Table 5) at 3T vs. 1.5T (both *p* < 0.0001, Table 4). As shown in Figs. 3 and 4, we explored the possible causes for the increased DGM volume at 3T by performing expert, manual segmentation of one scan each from a healthy control and a patient with MS. The manual segmentations were then overlaid on the automated segmentation maps. The 3T images provided a more accurate (and larger) contour of DGM structures. This was most likely related to improved contrast at the ventricular CSF-tissue interfaces, and to a lesser extent, the gray-white edges.

**Table 4:** Deep gray matter data: between group and between field strength comparisons

		MS group (*n* = 14)			NC group (*n* = 15)			p^a, b^ MS vs NC	
	*DGM structure*	*1.5T*	*3T*	*p (MS)* *1.5T vs. 3T*	*1.5T*	*3T*	*P (NC)* *1.5T vs 3T*	*1.5T*	*3T*
Volumes (ml)	Thalamus	14.4 (1.4) 11.7–16.3	14.7 (1.5) 12.6–16.7	0.003	15.2 (1.0) 12.9–16.3	16.0 (1.0) 13.6–17.1	<0.0001	0.05	0.02
	Caudate	6.2 (0.9) 4.9–7.8	6.5 (0.9) 5.0–8.2	0.0001	6.9 (0.8) 5.2–8.6	7.3 (0.9) 5.6–9.4	<0.0001	0.09	0.10
	Putamen	8.7 (0.7) 7.4–9.7	9.0 (0.7) 7.6–10.1	<0.0001	9.6 (0.8) 8.6–11.7	10.1 (0.9) 9.1–12.6	<0.0001	0.03	0.02
	Globus pallidus	2.9 (0.3) 2.4–3.4	3.1 (0.4) 2.6–3.9	0.0004	3.3 (0.3) 2.9–3.7	3.6 (0.3) 3.2–4.0	<0.0001	0.0006	0.0004
	Total DGM	32.2 (2.8) 26.4–37.1	33.4 (3.0) 28.1–38.6	<0.0001	35.0 (2.6) 30.9–40.2	37.0 (2.6) 32.8–42.8	<0.0001	0.02	0.01
Fractions	Thalamus	0.011 (0.0011) 0.0087–0.013	0.012 (0.00097) 0.0096–0.013	0.12	0.0116 (0.00058) 0.010–0.013	0.012 (0.00063) 0.011–0.013	0.0004	0.31	0.23
	Caudate	0.0049 (0.00057) 0.0037–0.0056	0.0051 (0.00058) 0.0040–0.0059	0.007	0.0053 (0.00046) 0.0040–0.0060	0.0055 (0.00053) 0.0043–0.0066	0.0004	0.20	0.30
	Putamen	0.0068 (0.00055) 0.0055–0.0078	0.0071 (0.00057) 0.0057–0.0080	0.0001	0.0074 (0.00051) 0.0065–0.0081	0.0077 (0.00053) 0.0068–0.0088	<0.0001	0.20	0.20
	Globus pallidus	0.0023 (0.00021) 0.0018–0.0026	0.0024 (0.00024) 0.0020–0.0028	0.0002	0.0025 (0.00017) 0.0023–0.0028	0.0028 (0.00013) 0.0025–0.0030	<0.0001	0.009	0.003
	Total DGM	0.0252 (0.0020) 0.0197–0.0282	0.0261 (0.0019) 0.0213–0.0280	0.002	0.0268 (0.0013) 0.0236–0.0290	0.0282 (0.0013) 0.0256–0.0299	<0.0001	0.11	0.07

Key: Data are mean (standard deviation) on first line; range on second line; Percent differences between 1.5T and 3T are shown in Table 5; DGM = cerebral subcortical deep gray matter; *MS* multiple sclerosis, *NC* normal controls; ^a^age adjusted; ^b^the differentiation (sensitivity) between MS and NC was similar for 1.5 versus 3T (all *p* > 0.05)

**Table 5:** Deep gray matter data: field strength comparisons: percent differences

	DGM structure	MS 1.5T vs. 3T	NC 1.5T vs. 3T
Volumes	Thalamus	2.7%	5.7%
	Caudate	4.8%	5.0%
	Putamen	4.1%	5.3%
	Globus pallidus	7.9%	10.4%
	Total DGM	3.9%	5.9%
Fractions	Thalamus	2.4%	5.2%
	Caudate	4.5%	4.5%
	Putamen	3.7%	4.8%
	Globus pallidus	7.5%	9.8%
	Total DGM	3.6%	5.4%
Normalized	Thalamus	3.2%	4.6%
	Caudate	5.3%	3.9%
	Putamen	4.6%	4.2%
	Globus pallidus	8.4%	9.2%
	Total DGM	4.4%	4.8%

Key: For each subject, the percent difference for 3T data minus 1.5T data was calculated, using the 1.5T data as the denominator. The averages of those percentages are shown for each group. DGM = cerebral subcortical deep gray matter; *MS* multiple sclerosis, *NC* normal controls; normalized = raw volume x SIENAX normalization factor

### Detection of DGM atrophy in MS vs. NC

A comparison of DGM volume and fraction in MS vs. NC is shown in Table 4. 3T showed slightly better differentiation of raw volumes (thalamus: *p* = 0.02; caudate: *p* = 0.10; putamen: *p* = 0.02; globus pallidus: *p* = 0.0004; total DGM: *p* = 0.01) than 1.5T (thalamus: *p* = 0.05; caudate: *p* = 0.09; putamen: *p* = 0.03; globus pallidus: *p* = 0.0006; total DGM: *p* = 0.02) for detecting DGM atrophy in MS vs. NC. Regarding the normalized volumes, atrophy was not detected in patients vs. NC in any structures at either field strength (*p* > 0.05) except for the globus pallidus at 3T (mean 4.4 vs. 5.0 ml, *p* < 0.05). Thus, thalamic and pallidal atrophy was detected slightly more definitively at 3T. Otherwise, the presence of atrophy in the other DGM structures was similarly detected at both 1.5T and 3T. Overall, these field strength differences in the ability to differentiate MS from NC were small (all *p* > 0.10). Comparing raw, fractional, and normalized volumes, there was slightly better sensitivity for detecting atrophy in the MS group for the raw volumes.

### Correlation between DGM and age or disability in the MS group

With the exception of the normalized putamen volume at 1.5T (*r* = −0.56, *p* = 0.04), DGM raw or normalized volumes and fractions at 1.5T or 3T did not correlate with age; the correlations were also not different between field strengths (all *p* > 0.05, data not shown). Regarding the correlation between DGM and EDSS scores, significant negative relationships were found with normalized volumes at 1.5T in the thalamus (*r* = −0.61, *p* = 0.02), globus pallidus (*r* = −0.53, *p* = 0.0498), and total DGM (*r* = −0.56, *p* = 0.04) and at 3T in the putamen (*r* = −0.56, *p* = 0.04). No significant correlations were found at either field strength between DGM raw volumes or fractions and EDSS scores (all *p* > 0.05, data not shown). The correlations were not different between field strengths (all *p* > 0.05, data not shown). Regarding the correlation between DGM and T25FW, with the exception of total DGM normalized volume (*r* = −0.55, *p* = 0.04), none of the comparisons reached significance (data not shown).

## Discussion

In this study, we explored the sensitivity and reliability of DGM volumes obtained from 1.5T vs. 3T and their clinical relevance. The first main finding was that volumes were generally higher at 3T. Secondly, 3T showed slightly better differentiation in the ability to detect atrophy in the thalamus and globus pallidus in MS vs. NC. Third, 3T showed slightly higher scan/re-scan reliability. We also noted that, regardless of field strength, volumes showed higher reliability than fractions, and total DGM volume was measured with higher reliability than the individual DGM nuclei. These data indicate that 1.5T and 3T are not interchangeable in measuring DGM volumes.

One clear finding was that raw volumes, fractions, and normalized volumes were higher at 3T vs. 1.5T across all DGM structures examined. In a previous study, volume biases were also detected based on field strength in comparing 1.5T to 3T using the FreeSurfer processing toolkit in 15 healthy elderly subjects; these occurred in either direction (not always higher at 3T) [22]. For example, the globus pallidus and thalamus showed significantly higher volumes at 1.5T but the amygdala was higher at 3T. Another study of whole brain volume measurements, comparing 1.5T and 3T MRI showed significant differences between platforms using the SIENAX toolkit [15]. Interestingly, this study found that the bias was in the opposite direction vs. the present study, with higher whole brain volumes measures from 1.5T. This was most likely related to an overestimation of brain volume at the sulcal-CSF interfaces at 1.5T due to partial volume averaging. In the present study, the DGM volumes from 3T may have been larger on the basis of more accurate detection of structure boundaries with CSF or adjacent white matter. Taken together, these results suggest that combining data across platforms and across field-strength introduces a bias that should be considered in the design of multi-site studies, such as clinical therapeutic trials.

In the detection of DGM atrophy in MS vs. NC in the present study, 3T showed slightly better differentiation. Similar findings were seen in a previous study in detecting hippocampal atrophy at 3T vs. 1.5T [23]. In that study, subjects who converted from mild cognitive impairment to Alzheimer disease within 3 years of baseline MRI showed significantly more atrophy in the cornu ammonis 1 region of the right hippocampus versus nonconverters at 3T but not at 1.5T. Another study, focusing on whole brain atrophy, showed a higher effect size for 3T in detecting brain atrophy in MS versus NC when compared to 1.5T [15]. These results parallel what has been shown regarding MS lesion detection at 3T and 7T, with an increase in the diagnostic yield in the detection of MS brain lesions compared to 1.5T [14, 24]; moreover, the brain lesion load at 3T showed a closer relationship to cognitive status than 1.5T [14]. These results underscore potential gains in sensitivity and validity in the MRI measurement of MS-related structural changes with ultra-high field strengths.

In the present study, we found atrophy of the DGM in patients with MS vs. NC in various nuclei. There are several mechanisms to consider in the pathogenesis of MS-related DGM atrophy. These include iron-deposition [25], oxidative stress [26], neurodegeneration [27], direct injury by the presence of DGM demyelinating lesions [28], and Wallerian degeneration due to damage of white matter tracts throughout the brain [29].

In a previous study, scan-rescan reliability for a variety of platforms at two field strengths was explored [30]. Their results showed that reliability of automatic brain morphometry obtained by FreeSurfer was generally higher with GE Signa Excite (1.5T) and Siemens Verio (3T) vs. Siemens Sonata (1.5T) and TrioTim (3T) acquisitions. The authors argued that, although TrioTim and Verio are both 3T MRI models from the same manufacture (Siemens), the results from the two machines differed significantly. Another group compared 3T Siemens scanners at seven sites to evaluate the difference between intra-scanner and inter-scanner reliability of lesion and atrophy-related MS volumetrics in a human phantom study using a variety of processing pipelines [31]; the authors showed that, despite protocol harmonization and the use of high-resolution sequences, a large degree of variability in the data was caused by inter-scanner effects. In the present study, we showed high scan-rescan reliabilities for both 1.5T and 3T in the assessment of DGM volumetry. In the measurement of DGM raw volumes, there was perhaps slightly higher reliability at 3T. However, for DGM fractions, there was perhaps somewhat higher reliability at 1.5T. Raw volumes showed slightly higher reliability than the other two normalized methods. Also, the most sensitivity in detecting DGM atrophy was observed with raw DGM volumes. This probably reflects the inaccurate estimation of ICV and the brain contour, necessary for normalization, due to higher susceptibility artifacts at 3T.

Several limitations of our study are worthy of comment. First, aside from the field strength difference, the two acquisitions differed on the scanner vendor, type of head coil, and use of parallel imaging (only at 3T). The voxel sizes also slightly differed. One should also consider the potential effect of DMTs on brain volume; first because of their partial but significant therapeutic effects on limiting the rate of atrophy in MS [3, 5, 6, 32]. Because most of our patients were receiving DMT, the generazibility of our results to untreated patients is not established by this study. Furthermore, there is the potential for individuals to show pseudoatrophy in the few months after their initiation of therapy (for some but not all DMTs) [33]. However, in the present study, none of patients had newly started their DMT in the previous 3 months, thus indicating that pseudoatrophy did not have a major effect on our results. In addition, the sample size was small and no longitudinal data were available to compare the rate of atrophy between the two acquisitions. We did not test other fully automated segmentation pipelines such as FreeSurfer and others that are available to measure DGM atrophy [31, 34–36]. Finally, our patient population was dominated by subjects with relapsing forms of MS, with only four people in our study having progressive forms of the disease. Thus, the generalizability of our results to the full MS spectrum would require further study. Thus, taken together, these caveats suggest other factors that could have influenced the differences we observed between the two MRI scan platforms.

## Conclusion

We conclude that MRI scan acquisition field strength should be considered in the design of longitudinal studies and multicenter clinical trials. Such differences may introduce bias in the obtained data and results. If such consistency cannot be maintained, statistically corrective modelling may be considered [37, 38].

## Abbreviations

CI: Confidence interval
CIS: Clinically isolated demyelinating syndrome
CSF: Cerebrospinal fluid
DGM: Cerebral subcortical deep gray matter nuclei
ICC: Intraclass correlation coefficient
ICV: Intracranial volume
MS: Multiple sclerosis
NC: Normal controls
NIfTI: Neuroimaging informatics technology initiative
T25FW: Timed 25-ft walk
